# Supplementary material for: Health Kiosks in Market: A qualitative study of factors influencing the transferability of the program to a target context in Kenya
Source: PLoS One. 2025 Oct 30;20(10):e0321357. doi: 10.1371/journal.pone.0321357 (PMC12574851; doi:10.1371/journal.pone.0321357)
Supplement: Attachment — Submitted filename: Review.docx [file pone.0321357.s001.docx]

**Recommendations for Authors**

Thank you for the opportunity to review the manuscript titled “Health Kiosks in market: A qualitative study of factors influencing the transferability of the program to a target context in Kenya”. While the topic is relevant and timely, I have several concerns regarding the manuscript’s current form. Below, I outline specific areas where the manuscript could be strengthened to improve its clarity, coherence, and overall contribution.

**Abstract**

- Please include the data collection period and the total number of participants.
- Kindly add 3-5 relevant keywords to support indexing and improve discoverability.

**Introduction**

- When referring to the disease burden or epidemiological situation, please specify the year to provide a clear temporal context. Also, consider updating the data if more recent statistics are available.
- The phrase “in Error! Reference source not found” appears to be a referencing error. Please review and correct the citation accordingly.

**Research Methods and Design**

**Setting**

- When stating the population size, please indicate the year the data refers to in order to ensure clarity and relevance.
- Including a map of the study area would enhance the reader’s understanding of the geographical context.

**Study Population and Sampling Strategy**

- Please specify the exclusion criteria.

**Results**

- Please provide the characteristics of participants for FGDs and IDIs to offer context for the findings.

**Discussion**

- Please include recommendations for further research to guide future work in this area.
- Kindly expand the discussion to provide deeper analysis or reflection on key findings and their implications for program transferability.

**Acknowledgment**

- Please include a note of appreciation to the study participants for their time and valuable contributions.

**Figure**

- Please ensure that Figure 1 is properly cited within the main text to maintain consistency and guide the reader.
- Figure 1 appears unclear and lacks sharpness. Please consider enhancing the resolution or quality to improve readability.
