# Supplementary material for: Health Kiosks in Market: A qualitative study of factors influencing the transferability of the program to a target context in Kenya
Source: PLoS One. 2025 Oct 30;20(10):e0321357. doi: 10.1371/journal.pone.0321357 (PMC12574851; doi:10.1371/journal.pone.0321357)
Supplement: Attachment — Submitted filename: Response to Reviewers.docx [file pone.0321357.s003.docx]

**Response to Reviewers**

I have managed to respond to each and every comment as advised.

| **Recommendations for Authors** | **Authors comment** | **page** |
| --- | --- | --- |
| **Additional Requirements** | | |
| Please ensure that your manuscript meets PLOS ONE's style requirements, including those for file naming. | The document has been redone and currently meets PLOS ONE’s style | **Across the whole document** |
| We note that you have indicated that there are restrictions to data sharing for this study. For studies involving human research participant data or other sensitive data, we encourage authors to share de-identified or anonymized data. However, when data cannot be publicly shared for ethical reasons, we allow authors to make their data sets available upon request. For information on unacceptable data access restrictions, please see | - **The author has sent the data to** [**Qualitative Data Repository**](https://qdr.syr.edu/) | **22** |
| If there are ethical or legal restrictions on sharing a de-identified data set, please explain them in detail (e.g., data contain potentially identifying or sensitive patient information, data are owned by a third-party organization, etc.) and who has imposed them (e.g., a Research Ethics Committee or Institutional Review Board, etc.). Please also provide contact information for a data access committee, ethics committee, or other institutional body to which data requests may be sent. |  |  |
| If there are no restrictions, please upload the minimal anonymized data set necessary to replicate your study findings to a stable, public repository and provide us with the relevant URLs, DOIs, or accession numbers. |  |  |
| Please review your reference list to ensure that it is complete and correct. If you have cited papers that have been retracted, please include the rationale for doing so in the manuscript text or remove these references and replace them with relevant current references. Any changes to the reference list should be mentioned in the rebuttal letter that accompanies your revised manuscript. If you need to cite a retracted article, indicate the article’s retracted status in the References list and also include a citation and full reference for the retraction notice. | I have added two references, among them Nos 5 and 21 having updated the literature and the type of analysis used. | **20 & 21** |
| **Reviewer #1** | | |
|  |  |  |
| The abstract lacks specificity in key findings. What exact factors (beyond generalities like "perceived usefulness") influenced transferability? Rewrite the results section of the abstract. | The results have been rewritten and the findings made specific | **2** |
| The introduction is overloaded with general background on NCDs; it could be summarized and added to with more Kenya-specific context and the rationale for choosing HEKIMA. | More Kenya specific has been added into the introduction section. | **3** |
| The flow between global issues and local intervention is not coherent | Inconsistencies redone on the instruction section. |  |
| In line 111, "Error! Reference source not found" suggests a missing figure or citation, indicating poor proofreading or reference management. | This is done |  |
| Lines 133–153 repeat participants in multiple ways and have too much redundancy in the sampling strategy | The study population and sampling strategy has been rewritten and the redundancy sorted | 6 & 7 |
| Line 181 states that "inductive content analysis" (ICA) was used, yet the coding process seems highly structured and deductive, given the PIET-T framework guidance. Clarify whether it was inductive or deductive | The has been revised as per the advice given | 8 |
| Lack of sufficient discussion on why certain elements supported or hindered transferability. For example, why did decision-makers support it in one county but have reservations in another | This is done |  |
| There are numerous grammatical errors, run-on sentences, and awkward phrasings throughout the manuscript. There are inconsistent references and punctuation (e.g., missing full stops and misplaced commas). The manuscript should be proofread thoroughly. | The manuscript has been proofread and is now okay for publication |  |
| **Reviewer #2** | | |
| **Abstract** | | |
| Please include the data collection period and the total number of participants. | This has been indicated on the abstract | **1** |
| Kindly add 3-5 relevant keywords to support indexing and improve discoverability. | This has been added | **1** |
| **Introduction** | | |
| When referring to the disease burden or epidemiological situation, please specify the year to provide a clear temporal context. Also, consider updating the data if more recent statistics are available | This is done |  |
| The phrase “in Error! Reference source not found” appears to be a referencing error. Please review and correct the citation accordingly. | This has been corrected |  |
| **Research Methods and Design** | | |
| When stating the population size, please indicate the year the data refers to in order to ensure clarity and relevance. | This has been stated | **6** |
| Including a map of the study area would enhance the reader’s understanding of the geographical context. | A map has been done |  |
| Please specify the exclusion criteria | **Exclusion criteria has been added** |  |
| **Results** | | |
| Please provide the characteristics of participants for FGDs and IDIs to offer context for the findings. | This has been provided in the biggening of the finding section | **9** |
| **Discussion** | | |
| Please include recommendations for further research to guide future work in this area. | This has been included as advised | **19** |
| Kindly expand the discussion to provide deeper analysis or reflection on key findings and their implications for program transferability. | This is done |  |
| **Acknowledgment** | | |
| Please include a note of appreciation to the study participants for their time and valuable contributions. | A note of appreciation to the participants added | **20** |
| **Figure** | | |
| Please ensure that Figure 1 is properly cited within the main text to maintain consistency and guide the reader. | This has been clearly cited |  |
| Figure 1 appears unclear and lacks sharpness. Please consider enhancing the resolution or quality to improve readability. | I have tried to enhance the resolution and sharpness of the figure | **Figures** |
| **Reviewer #2** | | |
| I am not sure, the researchers use the methodology of content analysis will help answer the question of factors influencing the transferability of the program to a target context in Kenya because the researcher used focus group and in-depth interviews. The study of factors should use quantitative methods, which should make the researcher's explanation more reliable. | This has been redone as advised. | **6** |
