# Supplementary material for: Health Kiosks in Market: A qualitative study of factors influencing the transferability of the program to a target context in Kenya
Source: PLoS One. 2025 Oct 30;20(10):e0321357. doi: 10.1371/journal.pone.0321357 (PMC12574851; doi:10.1371/journal.pone.0321357)
Supplement: Attachment — Submitted filename: Rebuttal letter_2.docx [file pone.0321357.s004.docx]

**Health Kiosks in Market: A qualitative study of factors influencing the transferability of the program to a target context in Kenya.**

**Dear Editor,**

Below is my response to each on the reviewer’s comments

| **Reviewers comment** |  |  |
| --- | --- | --- |
| **Reviewer 1** | **Author’s comments** | **Page** |
| While the authors mention developing 264 codes and merging them into 13 sub-themes, a brief mention of intercoder reliability will be beneficial. | While intercoder reliability is key in measuring consistency and replicability among others, it can only be applied when qualitative has been analyzed by more than one coder. This being a PhD, the researcher did not engage the services of other analysts hence it may not be possible to develop the reliability test. |  |
| Even though optional, a table summarizing the final themes/sub-themes under the PIET-T framework would aid readers' understanding and strengthen the presentation of results. | This has been included as advised | 8 |
| While the current description offers helpful qualitative details, it would be more informative to also include basic quantitative information, such as the number of participants in each group (e.g., how many FGD and IDI participants, by role or gender) to better contextualize the findings. | This information has been added in the beginning of the results section | 9-10 |
